# Molecular Characterization of the *Schistosoma mansoni* Zinc Finger Protein SmZF1 as a Transcription Factor

**DOI:** 10.1371/journal.pntd.0000547

**Published:** 2009-11-10

**Authors:** Marcela G. Drummond, Carlos E. Calzavara-Silva, Diego S. D'Astolfo, Fernanda C. Cardoso, Matheus A. Rajão, Marina M. Mourão, Elisandra Gava, Sérgio C. Oliveira, Andréa M. Macedo, Carlos R. Machado, Sérgio D. J. Pena, Gregory T. Kitten, Glória R. Franco

**Affiliations:** 1 Laboratório de Genética Bioquímica, Departamento de Bioquímica e Imunologia, Instituto de Ciências Biológicas, Universidade Federal de Minas Gerais, Belo Horizonte, Minas Gerais, Brazil; 2 Laboratório de Imunologia Celular e Molecular, Centro de Pesquisas René Rachou, FIOCRUZ, Belo Horizonte, Minas Gerais, Brazil; 3 Centro de Investigaciones en Bioquímica Clínica e Inmunología (CIBICI-CONICET), Departamento de Bioquímica Clínica, Facultad de Ciencias Químicas, Universidad Nacional de Córdoba, Córdoba, Argentina; 4 Laboratório de Imunologia de Doenças Infecciosas, Departamento de Bioquímica e Imunologia, Instituto de Ciências Biológicas, Universidade Federal de Minas Gerais, Belo Horizonte, Minas Gerais, Brazil; 5 Laboratório do Desenvolvimento do Coração e Matriz Extracelular, Departamento de Morfologia, Instituto de Ciências Biológicas, Universidade Federal de Minas Gerais, Belo Horizonte, Minas Gerais, Brazil; University of Queensland, Australia

## Abstract

**Background:**

During its development, the parasite *Schistosoma mansoni* is exposed to different environments and undergoes many morphological and physiological transformations as a result of profound changes in gene expression. Characterization of proteins involved in the regulation of these processes is of importance for the understanding of schistosome biology. Proteins containing zinc finger motifs usually participate in regulatory processes and are considered the major class of transcription factors in eukaryotes. It has already been shown, by EMSA (Eletrophoretic Mobility Shift Assay), that SmZF1, a *S. mansoni* zinc finger (ZF) protein, specifically binds both DNA and RNA oligonucleotides. This suggests that this protein might act as a transcription factor in the parasite.

**Methodology/Principal Findings:**

In this study we extended the characterization of SmZF1 by determining its subcellular localization and by verifying its ability to regulate gene transcription. We performed immunohistochemistry assays using adult male and female worms, cercariae and schistosomula to analyze the distribution pattern of SmZF1 and verified that the protein is mainly detected in the cells nuclei of all tested life cycle stages except for adult female worms. Also, SmZF1 was heterologously expressed in mammalian COS-7 cells to produce the recombinant protein YFP-SmZF1, which was mainly detected in the nucleus of the cells by confocal microscopy and Western blot assays. To evaluate the ability of this protein to regulate gene transcription, cells expressing YFP-SmZF1 were tested in a luciferase reporter system. In this system, the luciferase gene is downstream of a minimal promoter, upstream of which a DNA region containing four copies of the SmZF1 putative best binding site (D1-3DNA) was inserted. SmZF1 increased the reporter gene transcription by two fold (p≤0.003) only when its specific binding site was present.

**Conclusion:**

Taken together, these results strongly support the hypothesis that SmZF1 acts as a transcription factor in *S. mansoni*.

## Author Summary

Schistosomes are parasites that exhibit a complex life cycle during which they progress through many morphological and physiological transformations. These transformations are likely accompanied by alterations in gene expression, making genetic regulation important for parasite development. Here we describe a *Schistosoma mansoni* protein (SmZF1) that may act as a parasite transcription factor. These factors are key proteins for gene regulation. We have previously demonstrated that SmZF1 is able to bind DNA and that its mRNA is present at different stages during the parasite life cycle. In this study we aimed to define if this protein can function as a transcription factor in *S. mansoni*. SmZF1 was detected in the nucleus of adult male worms, cercariae and schistosomula cells. It was not, however, observed in female cells, suggesting it to be gender specific. We used mammalian cells expressing recombinant SmZF1 to analyze if SmZF1 protein is able to activate/repress gene transcription and demonstrated that it increased the expression of a reporter gene by two-fold. The results obtained confirm SmZF1 as a *S. mansoni* transcription factor.

## Introduction

Schistosomiasis is a disease caused by trematode worms, mainly *Schistosoma mansoni*, *S. haematobium* and *S.japonicum*. According to World Health Organization, this parasitic disease affects 200 million people throughout the world [1]. Although the level of schistosome-associated morbidity is unclear, some recent studies have demonstrated that the illness is a more serious problem than it was previously thought to be [2],[3]. Therefore, emphasis should be focused on mechanisms that could not only prevent, but also cure schistosomiasis. A useful approach to fight the disease should include infrastructure and educational components, as well as the development of vaccines and new drugs [4]. Luckily we are living a special moment, with the recent publication of both *S. mansoni* [5] and *S. japonicum* [6] genomes, which will bring to the scientific community an enormous amount of data to be mined in the search for new therapeutic targets and vaccine development. Lastly, additional effort should also be dedicated to studies regarding the biology and development of the parasite.

During its life cycle, *S. mansoni* is exposed to different environmental conditions: water, intermediate molluscan host, and a definitive vertebrate host. As a consequence, this parasite suffers many transformations in its morphology and physiology, and, as such, represents an interesting but challenging biological system to investigate gene regulation processes [7]–[9]. A variety of publications have focused on the identification and characterization of *S. mansoni* stage-, tissue- and sex-specific/abundant proteins and their coding genes [10]–[14], which may uncover hidden aspects of parasite biology and thus provide useful leads for the development of novel intervention strategies [7]. In a primary analysis of the *S. mansoni* transcriptome, Verjovski-Almeida and colleagues suggested that the number of differentially expressed genes could reach as many as 1000 for each stage [15]. In more recent publications, in which analyses of gene expression were carried out using microarray, SAGE (Serial Analysis of Gene Expression) and proteomic experiments, the authors confirmed a number of sex- and stage-specific, differentially expressed genes [8], [16]–[26].

In order to better understand the transcriptional regulation of *S. mansoni* genes, it is necessary to identify new transcription factors, coactivators/corepressors and chromatin remodeling factors that control this molecular process, along with regulatory elements in the promoter region of genes [9]. Several efforts to describe new transcription factors in this parasite have been made [27]–[31], but given the complexity of its life cycle there are still many components to be discovered and characterized. Zinc finger motifs are found in several proteins amongst eukaryotic organisms and are key proteins for transcription regulation [32]–[34]. SmZF1 is a *S. mansoni* 19 kDa protein (GenBank accession number AAG38587) containing three C_2_H_2_ type zinc finger motifs. Its cDNA was casually isolated from an immune screening of a *S. mansoni* adult worm lambda gt11 expression library using an anti-tegumental serum. The transcript coding for SmZF1 was also detected by PCR amplification in egg, cercaria, schistosomulum and adult worm cDNA libraries, suggesting that the protein is essential for metabolism during different stages of the parasite life cycle [35]. In a previous work, we used a recombinant SmZF1 protein in EMSA experiments to investigate its binding capacity/specificity for DNA and RNA oligonucleotides. SmZF1 was found to bind both double and single-stranded DNA, as well as RNA oligonucleotides, but with about 10-fold lower affinity. Although we noticed that SmZF1 recognized DNA and RNA oligonucleotides not containing putative target sites, the protein bound preferentially to the ones containing the sequence 5′-CGAGGGAGT-3′ (oligonucleotide D1-3DNA). Furthermore, unrelated oligonucleotides were not able to abolish this interaction. Taken together, these initial results suggested that SmZF1 may act as a putative transcription factor in *S. mansoni* [36].

In order to better characterize the biological function of the SmZF1 protein, in this study we proposed to: (i) verify the subcellular localization of SmZF1 in the cells of *S. mansoni*, as well as in mammalian COS-7 cells expressing a recombinant YFP (Yellow Fluorescent Protein)-SmZF1 protein; (ii) test the ability of SmZF1 to activate or repress gene transcription. The results described herein define SmZF1 as a *S. mansoni* nuclear protein capable of activating gene transcription.

## Materials and Methods

### Anti-SmZF1 specific antibodies

In order to obtain anti-SmZF1 antibodies, the MBP (Maltose Binding Protein) portion of a MBP-SmZF1 recombinant protein [36] was cleaved using Factor Xa protease (New England Biolabs, Ipswitch, MA, USA). The cleavage reaction was carried out for 48 h at 4°C in a 1∶25 enzyme: protein proportion. After digestion and fractionation by electrophoresis, a Coomassie blue-stained protein band (450 µg), representing the SmZF1 portion of the recombinant protein was excised from a 10% SDS-PAGE, homogenized with PBS (Phosphate Buffered Saline – 130 mM NaCl, 2 mM KCl, 8 mM Na_2_HPO_4_, 1 mM KH_2_PO_4_), then emulsified with Complete Freund Adjuvant and used for the primary intramuscular injection into a rabbit or with Incomplete Freund Adjuvant for the two subsequent boosts (15 and 30 days after the first immunization). Pre-immune serum was obtained before the first immunization and rabbit serum containing anti-SmZF1 antibodies was collected 15 days after the third immunization.

### *S. mansoni* immunohistochemistry assays

*S. mansoni* adult worms used in this study were recovered from perfused mice. Lung-stage schistosomula were prepared according to Harrop and Wilson [37]. Cercariae were obtained from *Biomphalaria glabrata* by exposing the infected snails to light for 2 h to induce shedding of parasites.

Sections of Omnifix (AnCon Genetics Inc., Melville, NY, USA) fixed, paraffin-embedded adult male or female worms were deparaffinized using xylol, hydrated with an ethanol series, washed in PBS and then incubated in a blocking solution (0.05% Tween 20, 1% w/v BSA (Bovine Serum Albumin) in PBS pH 7.2) overnight at 4°C. Samples were reacted for 1 h with either the anti-SmZF1 or a control, pre-immune rabbit serum, both diluted 1∶30 in 10x diluted blocking solution. Sections were then washed in PBS and reacted for 1 h with a 1∶400 diluted goat anti-rabbit IgG-Cy-5 conjugate (Jackson Immunoresearch Laboratories Inc., West Grove, PA, USA) in 10x diluted blocking solution, which also contained Alexa Fluor 488 phalloidin (Invitrogen, Carlsbad, CA, USA) diluted 1∶100 to stain actin microfilaments (except for adult male worms). Afterwards, samples were washed, incubated for 10 min with 1∶3000 diluted propidium iodide (Sigma-Aldrich, St. Louis, MO, USA) in 10x diluted blocking solution to stain nuclei and then washed with PBS.

For experiments using cercariae and lung-stage schistosomula, a whole-mount protocol was chosen. Omnifix fixed cercariae were treated with a permeabilizing solution (0.1% Triton X-100, 1% w/v BSA and 0.1% w/v sodium azide in PBS pH 7.4) for 3 h at 4°C under constant agitation. Subsequent immunostaining steps used the same solution and condition. Samples were incubated overnight with the anti-SmZF1 antibody diluted 1∶90, washed several times and reacted for 4 h with the goat anti-rabbit IgG-Cy-5 conjugate diluted 1∶1200 in solution containing Alexa Fluor 488 phalloidin (1∶500). The cercariae were then incubated for 20 min with propidium iodide diluted 1∶6000 and washed once more. The schistosomulum immunohistochemistry assays were carried out as with cercaria, with the following modifications: lung stage schistosomula were treated with permeabilizing solution overnight and then incubated with the anti-SmZF1 antibody (1∶90) for 2 h. The secondary antibody was used at a 1∶1000 dilution, and the phalloidin at a 1∶100 dilution for 2 h.

Samples (adult male and female worms, schistosomula and cercariae) were prepared with a mounting solution (90% glycerol, 10% tris-HCl 1 M, pH 8.0) and the fluorescence images were captured with a Carl Zeiss LSM 510 META confocal microscope using a 63x oil-immersion objective lens in the Center of Electron Microscopy (CEMEL-ICB/UFMG). Images were analyzed with Zeiss LSM Image Browser software and edited with Adobe Photoshop CS.

All research protocols involving mice used in the course of this study were reviewed and approved by the local Ethics Committee on Animal Care at Universidade Federal de Minas Gerais (CETEA – UFMG N° 023/05).

### RNA extraction and Real-Time PCR

Adult worms recovered from perfused mice were manually separated and pooled according to their sex. Total RNA of both male and female worms was extracted using Trizol reagent (Invitrogen) and treated with DNase using Ilustra RNAspin Mini RNA Isolation Kit (GE Healthcare, Waukesha, WI, USA) according to the manufacturer's instructions. RNA was then quantified using a NanoDrop Spectrophotomer ND-1000 (Thermo Scientific, Waltham, MA, USA). cDNA was synthesized using 0.3 to 1.0 µg total RNA and Superscript III First-Strand Synthesis SuperMix for qRT-PCR (Invitrogen) according to the manufacturer's protocol. For q-PCR reactions, the primers SmZF1_real2_forw (5′–ACTTCTCTCAGAAATCCAGCCT–3′) and SmZF1_real2_rev (5′–TGGAGAGGATTATACAATCTGGTT–3′) were used at a 600 nM initial concentration. The *S. mansoni* glyceraldehyde 3-phosphate dehydrogenase (GAPDH) gene (primers GAPDH_forw 5′–TCGTTGAGTCTACTGGAGTCTTTACG–3′ and GAPDH_rev 5′–AATATGAGCCTGAGCTTTATCAATGG–3′) was used as an endogenous control in order to normalize relative amounts of total RNA. GAPDH primers were used at a 900 nM initial concentration. The amplicon sizes were 96 bp and 65 bp for SmZF1 and GAPDH, respectively. q-PCR reaction mixtures consisting of 2.5 µl of cDNA, 12.5 µl of Power SYBR Green PCR Master Mix (Applied Biosystems, Foster City, CA) and 5 µl of each primer in a total volume of 25 µl were added to 48-Well Optical Reaction Plates for amplification and quantification in a StepOne Real-Time PCR System (Applied Biosystems). Each q-PCR run was performed with two internal controls in order to assess both potential genomic DNA contaminations (i.e., no reverse transcriptase added in the cDNA synthesis) and purity of the reagents used (i.e., no cDNA added). Dissociation curve standard analyses were performed at the end of each assay to certify the specific amplifying of targets. For each set of primers, both male and female conditions (including negative controls) were run in three technical replicates. The experiment was repeated two times (biological replicates) and the delta-delta Ct method [38] was used in order to make a relative quantification comparing male and female transcript levels. Due to the nonparametric distribution of data, statistical analysis of delta-delta Ct values was performed using the Mann-Whitney U-test with significance set at P<0.05.

### Plasmid constructs and COS-7 cells culture

The SmZF1 cDNA was PCR amplified in a reaction mixture prepared in a 50 µL final volume containing 25 ng of template DNA, 0.2 pmol µL^−1^ of each primer (SmZF1-start-Xba: 5′–CAGTCTAGAACTTTAACTATGGAATT-3′ and SmZF1-stop-Apa: 5′-CAGGGGCCCCATCCGGAAAGGCTTGAGA-3′, or SmZF1-start-Sac: 5′-CAGGAGCTCACTTTAACTATGGAATT-3′ and SmZF1-stop-Hind: 5′-CAGAAGCTTCATCCGGAAAGGCTTGAGA-3′), 200 mM dNTPs and 5 U of Taq DNA polymerase (Phoneutria, Belo Horizonte, MG, Brazil) in the appropriate buffer (50 mM KCl, 10 mM Tris-HCl pH 8.4, 0.1% Triton X-100, 1.5 mM MgCl_2_). The fragments obtained were double-digested with *Xba*I and *Apa*I or *Sac*I and *Hin*dIII restriction enzymes (New England Biolabs) and purified using a Wizard SV Gel and PCR Clean-up System (Promega, Madison, WI, USA) following the manufacturer's instructions. The fragments were then inserted, respectively, into the commercial vectors pCDNA4/TO/myc-His (Invitrogen) or pEYFP-c1 (Clontech, Mountain View, CA, USA), generating the constructions pCDNA4-SmZF1 and pEYFP-SmZF1, which express the recombinant proteins SmZF1-myc tag and YFP-SmZF1, respectively. In addition, the viral thymidine kinase (tk) promoter region was inserted (*Nhe*I/*Bgl*II) into the commercial vector pGL3-basic (Promega), generating the vector pGL3-tk-luc, with the luciferase (luc) reporter gene under control of the thymidine kinase promoter. Subsequently, an oligonucleotide containing four repetitions of the putative SmZF1 DNA binding site, D1-3DNA [36], was inserted (*Kpn*I/*Nhe*I) upstream of the minimal tk promoter, producing the vector pGL3-zf-tk-luc. The oligonucleotide sequence was as follows: 5′-CAGGAAACAGCTATGACCGG**CGAGGGAGT**GATCGG**CGAGGGAGT**GATCGG**CGAGGGAGT**GATCGG**CGAGGGAGT**GTCGTGACTGGGAAAACCCTGGCG-3′ (specific binding sites D1-3DNA are indicated in bold).

Ligation products were used to transform the *E. coli* DH5*a* strain and the rescued plasmids were sequenced using 10 pmol of appropriate primers (for constructions based on pCDNA4/TO/myc-His: CMV-fow 5′-CGCAAATGGGCGGTAGGCGTG–3′ and BGH-rev 5′-TAGAAGGCACAGTCGAGG–3′, for constructions based on pEYFP-c1: YFP-fow 5′-TTTTGCTCACAGGTTCT–3′ and YFP-rev 5′-GCCGTAGGTGGCATCGCC–3′, for constructions based on pGL3-basic: GLprimer2 5′-CTTTATGTTTTTGGCGTCTTCCA-3′ and RVprimer3 5′-CTAGCAAAATAGGCTGTCCC-3′), 4 µL of DYEnamic ET Dye Terminator Kit – MegaBACE (GE Healthcare) and 300 ng of DNA. The sequencing products were analyzed in the MegaBACE 1000 DNA Sequencer (GE Healthcare).

The above plasmid constructs were used either to transfect or co-transfect COS-7 cells using Lipofectamine™ 2000 Transfection Reagent (Invitrogen), according to the manufacturer's protocol. COS-7 cells were maintained at 37°C, 5% CO_2_ in Dulbecco's modified Eagle's medium (Invitrogen) supplemented with 10% fetal bovine serum and 1% glutamine (Invitrogen).

### COS-7 cells fluorescence microscopy and Western blot assays

The plasmids pEYFP-c1 (control) or pEYFP-SmZF1 were transfected (as above) into COS-7 cells for transient protein expression studies. Forty-eight hours after transfection the culture medium was carefully removed and cells were fixed (15 min) with 3% paraformaldehyde in PBS, washed and then quenched using PBS plus 10 mM NH_4_Cl (10 min). Cells were washed three times with PBS and incubated for 7 min with 0.1% Triton X-100. After another wash in PBS, COS-7 cells nuclei were stained (4 min) with 5 µL of 1 mM Hoechst 33342 dye (Sigma-Aldrich). The fluorescence was directly observed using a confocal microscope (Carl Zeiss LSM 510 META, 200x) equipped with a Photometrics Quantix CCD camera controlled by MetaMorph imaging software (MDS Analytical Technologies, Downingtown, PA, USA).

For Western blot assays, COS-7 cells (0.5×10^6^) transfected either with pCDNA4-SmZF1 or pEYFP-SmZF1 and control cells transfected either with pEYFP or pCDNA were washed and resuspended in 200 µL of cold TNE (150 mM NaCl, 50 mM Tris-HCl pH 7.5 and 1 mM ethylenediaminetetraacetic acid (EDTA)). A 50 µL aliquot of cells was centrifuged (700 g, 4 min, 4°C) and the pellet resuspended in 50 µL of 2x SDS gel-loading buffer (100 mM Tris-HCl pH 6.8, 200 mM dithiothreitol, 4% SDS, 0.2% bromophenol blue, 20% glycerol) and boiled for 5 min, generating the total extract. The remaining 150 µL of cells was centrifuged (700 g, 4 min, 4°C) and the pellet resuspended in 40 µL of lysis buffer (10 mM Tris-HCl pH 7.5, 10 mM NaCl, 2 mM MgCl_2_, 1 mM phenylmethylsulphonylfluoride (PMSF), one dissolved tablet of Complete Protease Inhibitor Cocktail (Roche, Basel, Switzerland), 1 mM Na_3_VO_4_ and 1 mM NaF) plus 100 µL of 1% Nonidet P-40 (Sigma-Aldrich) in 50 mM Tris-HCl pH 7.5. Samples were incubated in an ice bath for 10 min and centrifuged (700 g, 4 min, 4°C). Ninety-five microliters of 5x SDS gel-loading buffer was added to the supernatant, which was boiled for 5 min, generating the cytoplasmic fraction. The pellet was washed twice with cold TNE, centrifuged (700 g, 4 min, 4°C), resuspended in 50 µL of 2x SDS gel-loading buffer and boiled for 15 min, generating the nuclear fraction. COS-7 total, cytoplasmic and nuclear extracts, normalized at equal volume percentage, were separated using 10% SDS-PAGE and blotted (2 h, 20 mA) onto nitrocellulose membranes (Whatman GmbH, Dassel, Germany) using a semi-dry blot system (GE healthcare). Antibody reactions were performed as described by Koritschoner and colleagues [39]. Briefly, membranes were blocked overnight in TBS (25 mM Tris-HCl pH 7.4, 137 mM NaCl, 5 mM KCl, 0.6 mM Na_2_HPO_4_, 0.7 mM CaCl_2_, 0.5 mM MgCl_2_) plus 1 mM EDTA, 1 mM Na_3_VO_4_, 0.05% Tween-20 and 3% BSA followed by two washes with 100 mM Tris-HCl pH 8.0, 200 mM NaCl, 0.2% Tween-20 (wash buffer). Samples were reacted with anti-myc, anti-GFP or anti-c-erbB-2 (1∶1000) peroxidase conjugated antibodies (BD Biosciences, Franklin Lakes, NJ, USA) in blocking buffer for 1 h. Subsequently, blots were washed and developed with ECL enhanced chemiluminescence reagents (GE Healthcare) and exposed to X-ray film. The exclusively cytoplasmic protein c-erbB-2 was used as a quality control for extracts.

### Electrophoretic mobility shift assay

For the electrophoretic mobility shift assay (EMSA), 20 pmol of the D1-3DNA oligonucleotide (5′-CGAGGGAGT-3′) was incubated with 1 µg of the total extract of COS-7 cells transfected with plasmids pEYFP-c1 (control) or pEYFP-SmZF1. Extracts were produced as follows: cells (0.5×10^6^) were washed in PBS and resuspended in 100 µL of TDGK solution (20 mM Tris-HCl pH 7.5, 2 mM dithiothreitol, 400 mM KCl, 5 µg/ml leupeptin, 5 µg/ml aprotinin, 20% glycerol, 0.5 mM PMSF, 1 mM Na_3_VO_4_). Samples were maintained on ice for 30 min, centrifuged (15000 g, 20 min, 4°C) and then the supernatant was collected. Protein concentrations were measured and normalized as previously described [36]. The extract/DNA binding reactions were carried out in a final volume of 15 µL of binding solution (4 mM Tris-HCl pH 8.0, 40 mM NaCl, 1 mM ZnSO_4_, 4 mM MgCl_2_, 5% glycerol) for 15 min at 4°C. For supershift reactions, the DNA/extracts mixture was incubated, as above, with 1 µL of anti-GFP or 2 µL of anti-SmZF1 antibodies. After incubations, samples were fractionated in a 4% non-denaturing polyacrylamide gel in TBE buffer (89 mM Tris-borate pH 8.0, 2 mM EDTA), at a constant 25 mA at 4°C, to separate the bound complex from the free oligonucleotides. The resulting gels were stained with VISTRA Green DNA specific dye (GE Healthcare), according to the manufacturer's protocol.

### Transient co-transfections and luciferase activity assays

Plasmid DNA co-transfections of COS-7 cells were carried out in 24-well plates (Corning Inc., Corning, NY, USA). The day before transfection, 8×10^4^ COS-7 cells were plated in 0.5 ml of medium/well. For each well, 2 µl of LipofectamineTM 2000 Transfection Reagent were mixed with 1.2 µg of the plasmid DNA of interest and 300 ng of TK-Renilla reporter plasmid in serum-free Opti-MEM (Invitrogen) to allow the formation of DNA-LipofectamineTM 2000 Transfection Reagent complexes. The complexes were added to the respective wells and mixed by gently rocking the plate back and forth. Cells were incubated in a 5% CO_2_ incubator at 37°C for 48 h and then lysed with 60 µl of reporter lysis buffer (Promega). Luciferase activity (Relative Light Units – RLU) was assayed with 20 µl of lysate and 80 µl of luciferase assay reagent (Promega) in a TD20/20 luminometer (Promega) using a 10 s measurement period. Each transfection was performed in triplicate. Transfection efficiency was normalized to TK-Renilla luciferase reporter plasmid. Statistical analysis of the data was carried out with Minitab Version 1.4 using Student's t test with Welch's correction. Only p values<0.05 were considered as significant.

## Results

### SmZF1 has a nuclear localization at diverse *S. mansoni* developmental stages

*SmZF1* (GenBank accession AF316828) was initially identified during a screen of an adult worm *S. mansoni* cDNA library [35]. Although it has also been detected in cDNA libraries of other developmental stages of this parasite (i.e., egg, 3 h schistosomulum and cercaria), the biological function of the protein coded by this gene remains to be elucidated. The SmZF1 protein contains three C_2_H_2_-type zinc finger motifs and binds specific DNA oligonucleotides, as do similar nuclear proteins involved in gene transcriptional regulation [35],[36]. Therefore, to investigate whether SmFZ1 is present in the nucleus, where it could act as a transcription factor, we decided to verify its subcellular localization at diverse *S. mansoni* life stages.

**Figure 1:**
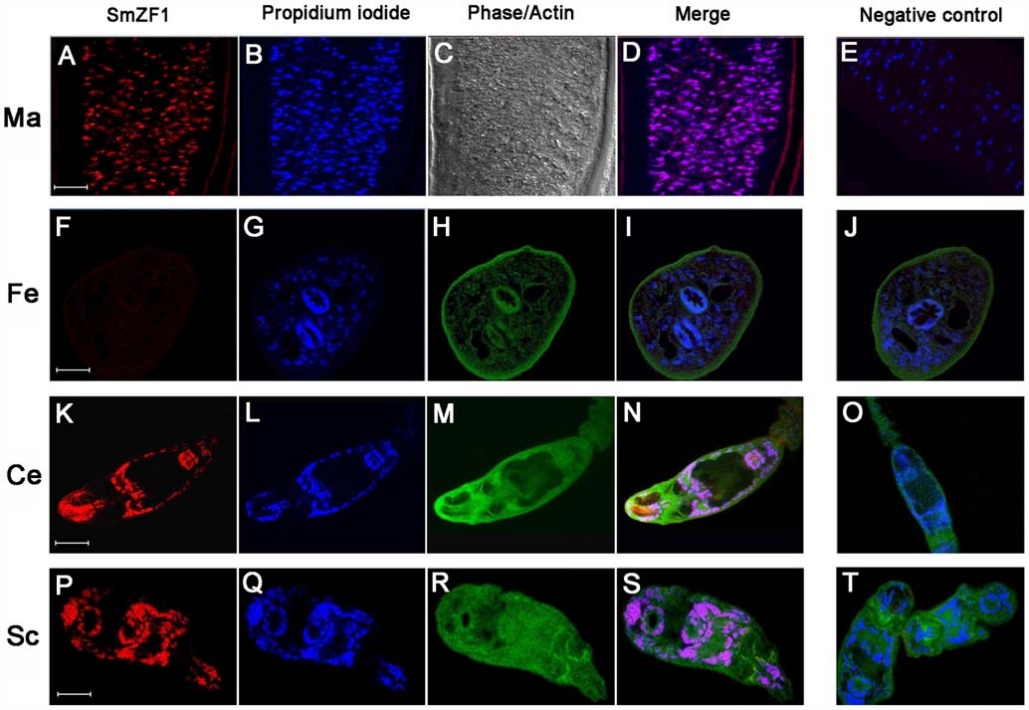
Native SmZF1 displays a nuclear localization at diverse stages of *S. mansoni* development. Fixed parasites were incubated with a rabbit anti-SmZF1 antibody, and then with a Cy-5 conjugated anti-rabbit IgG in a solution containing Alexa Fluor 488 phalloidin to stain actin microfilaments (phalloidin staining was not used with adult male worms). Samples were incubated with propidium iodide to visualize cells nuclei. Fluorescent images were obtained using a 63x oil-immersion objective lens and confocal microscopy (Carl Zeiss LSM 510 META). Images were analyzed with the Zeiss LSM Image Browser software and edited with Adobe Photoshop CS. To help distinguish the individual fluorescence signals, the original fluorescence colors were digitally modified. In the figure, fluorescence from Cy-5 is shown in red, propidium iodide in blue and phalloidin-Alexa fluor 488 in green. The merged blue and red produces a pink color. For adult male worms a phase contrast image is also presented. The following developmental stages were assayed: adult male (A–D) and female (F–I) worms, cercaria (K–N) and schistossomulum (P–S). Rabbit pre-immune serum was used as a control and was negative in all samples (E, J, O and T). Bars = 20 µm.

We carried out *in situ* immunohistochemistry experiments using an anti-SmZF1 antibody on *S. mansoni* collected at various stages during its life cycle. Western blot assays using the recombinant SmZF1 protein previously separated from its MBP portion, as well as fractionated extracts form adult worms revealed that this polyclonal antibody is specific to SmZF1 (Supporting information, Figure S1). The immunohistochemistry assays showed that SmZF1 protein localizes in the cells nuclei of adult male worms (Figures 1A–D), cercariae (Figures 1K–N) and lung stage schistosomula (Figures 1P–S). Although we have performed three different experiments in which we analyzed various paraffin sections of female adult worms, the protein could not be detected in this stage using this technique (Figures 1F–I and Supporting information, Figure S2). No SmZF1 staining was observed in the negative controls (Figures 1E, J, O, T) in which only the rabbit pre-immune serum was used. These results suggest that SmZF1 is a *S. mansoni* protein present in the nuclei of cells from diverse developmental stages where it may act as a transcription factor. Plus, *SmZF1* expression might be sex-specific since it could not be detected in adult female worms.

### The SmZF1 mRNA is equally expressed in adult male and female worms

We were unable to confirm the results from the immunohistochemistry experiments showing differences in expression of SmZF1 between male and female by Western blot, since nuclear protein extraction from single sex pooled *S. mansoni* worms did not provide sufficient material necessary for SmZF1 detection. Therefore, we decided to verify gene expression by comparing the transcript levels between adult male and female worms. Total RNA extraction was performed in separate pools of male or female worms and q-PCR analyses were carried out using primers specifically designed for SmZF1 amplification. We detected no difference in *SmZF1* expression (p = 0.22) between male and female worms when comparing the amplification profile, indicating that the SmZF1 mRNA is equally present in both genders (data not shown). These results suggest that although the *SmZF1* gene is transcribed in female worms, a post-transcriptional regulatory mechanism could be occurring to block SmZF1 protein production in adult female worms.

### SmZF1 goes to the nucleus when expressed in mammalian cells

**Figure 2:**
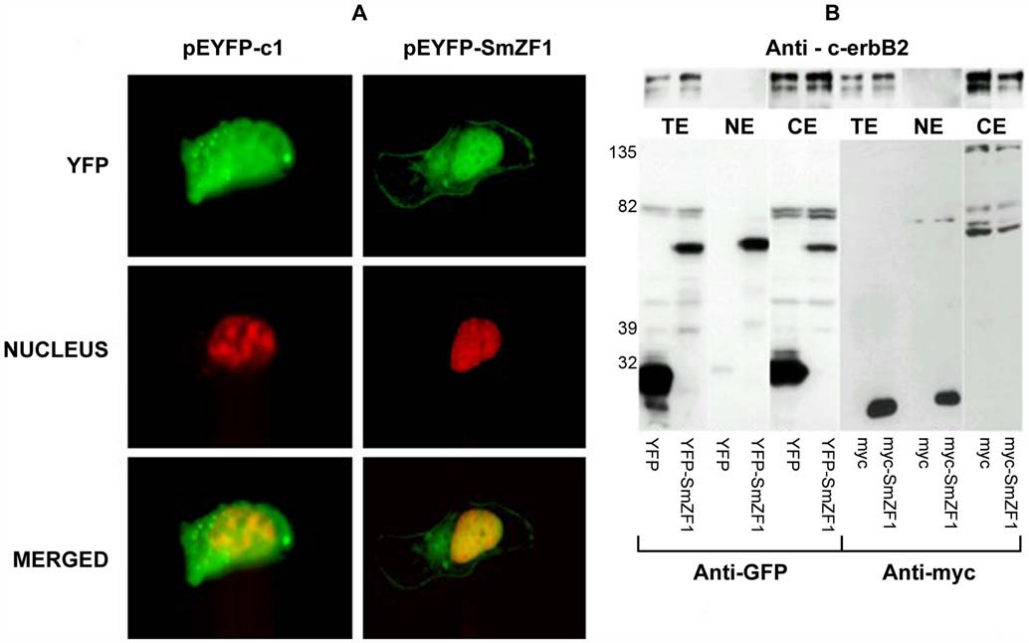
SmZF1 has a nuclear localization in COS-7 cells. (A) COS-7 cells were transfected either with pEYFP-c1 (control) or pEYFP-SmZF1 plasmids for transient expression of the proteins YFP or YFP-SmZF1. After 48 h the cells were fixed with paraformaldehyde, quenched, permeabilized and the cells nuclei stained using Hoechst 33342 dye. Images were directly captured at 200x magnification using Carl Zeiss LSM 510 META. In order to obtain a clearer visualization of fluorescent signals in merged images, the original yellow fluorescence of YFP and blue fluorescence of Hoechst were modified to green and red, respectively, using Adobe Photoshop CS2. (B) SmZF1-transfected and control COS-7 cells were used to generate the total (TE), cytoplasmic (CE) and nuclear (NE) extracts. The extracts were submitted to 10% SDS-PAGE and blotted onto nitrocellulose membranes. Membranes were blocked overnight and samples were reacted with anti-myc, anti-GFP or anti-c-erbB-2 peroxidase conjugated antibodies (1∶1000). Subsequently, blots were washed and developed with ECL enhanced chemiluminescence reagents and exposed to X-ray films. The exclusively cytoplasmic human oncoprotein c-erbB-2 was used as a quality control for extracts. YFP can be visualized as an approximately 30 kDa protein and the recombinant protein YFP-SmZF1 as an approximately 50 kDa protein.

After demonstrating the nuclear localization of SmZF1 in *S. mansoni* cells, the next step in the protein characterization was to heterologously express it in a mammalian system to test its ability to activate the transcription of a reporter gene. To accomplish this, we initially transfected COS-7 cells with the pEYFP-SmZF1 construction and forty-eight hours after transfection, we verified the presence of the YFP-SmZF1 recombinant protein mainly in the cells nuclei using fluorescence microscopy. However, a low level of fluorescent staining remained in the cytoplasm. In some cases, the protein was also visualized as fibrous material in the perinuclear region, probably associated with the cytoskeleton or Golgi complex. The YFP protein (negative control) was visualized diffusely distributed throughout the cells area (Figure 2A).

Since part of the fusion protein still remained in the cytoplasm of the cells, a second construction lacking YFP (SmZF1-myc tag) was used to confirm the SmZF1 nuclear localization in mammalian cells. Western blot assays using equal amounts of total, cytoplasmic and nuclear extracts of COS-7 cells expressing the proteins YFP, YFP-SmZF1 or SmZF1-myc tag were performed. Fractions were analyzed using either anti-GFP (which also recognizes YFP) or anti-myc antibodies (Figure 2B). The results corroborated those obtained by fluorescence microscopy (Figure 2A), showing that YFP-SmZF1 is present in both nuclear and cytoplasmic extracts, with a slight enrichment of the protein in the nuclear extract (Figure 2B). However, the recombinant protein SmZF1-myc tag is only present in the nuclear COS-7 extract, suggesting that YFP may be interfering in the transport of the fusion protein to the nucleus. The quality of the fractionation was confirmed by the localization of the cytoplasmic protein c-erbB-2 in the total and cytoplasmic fractions only (Figure 2B).

### YFP- SmZF1 is able to bind specific DNA oligonucleotides and activate transcription in COS-7 cells

In previous experiments using purified recombinant SmZF1 protein expressed in bacteria, we demonstrated the nucleic acid binding ability and specificity of SmZF1, its preference for DNA as compared to RNA, and its putative best DNA binding sequence (D1-3DNA) [36].

**Figure 3:**
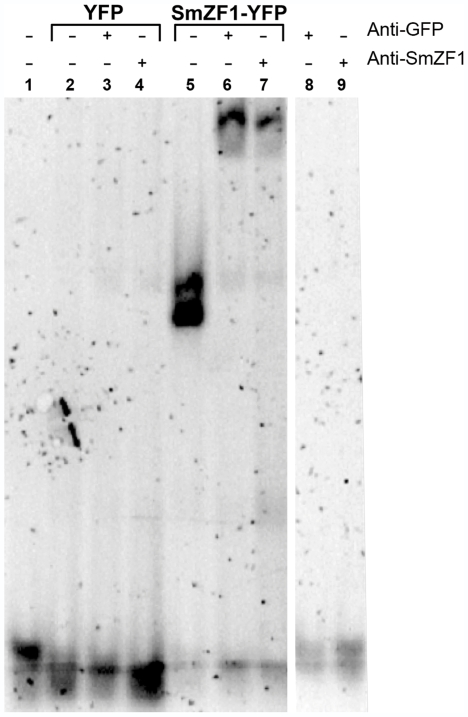
SmZF1 heterologously expressed in COS-7 cells is able to bind to its target oligonucleotide. For EMSA experiments, SmZF1 target oligonucleotide D1-3DNA was incubated with no extract (lane 1) or total extracts of COS-7 cells expressing the proteins YFP (lanes 2–4) or YFP-SmZF1 (lanes 5–7). For supershift reactions, the mixture DNA/extracts was pre-incubated with anti-GFP (lanes 3 and 6) or anti-SmZF1 antibodies (lanes 4 and 7). As negative controls, the anti-GFP and anti-SmZF1 antibodies were also incubated with the oligonucleotide in the absence of cell extracts (lanes 8 and 9, respectively). The resulting 4% PAGE was stained with VISTRA Green DNA specific dye for visualization of bands.

To verify whether the recombinant protein YFP-SmZF1 expressed in mammalian COS-7 cells was able to interact with D1-3DNA binding site in a manner comparable to its recombinant prokaryotic counterpart, EMSA assays were performed. Total extracts of COS-7 cells transfected with either pEYFP-c1 or pEYFP-SmZF1, expressing YFP or YFP-SmZF1, respectively, were incubated with the D1-3DNA oligonucleotide. To confirm the SmZF1/D1-3DNA interaction, supershift assays using anti-GFP and anti-SmZF1 antibodies were also performed. Extracts of cells expressing the YFP-SmZF1 recombinant protein were able to shift the oligonucleotide migration in the gel (Figure 3, lane 5). Additionally, both anti-GFP and anti-SmZF1 antibodies were able to supershift D1-3DNA migration, confirming that the YFP-SmZF1 protein was responsible for the oligonucleotide binding (Figure 3, lanes 6 and 7). Extracts of cells expressing only the YFP protein (Figure 3, lanes 2–4), as well as anti-GFP and anti-SmZF1 antibodies (Figure 3, lanes 8 and 9), were not able to shift the D1-3DNA migration.

**Figure 4:**
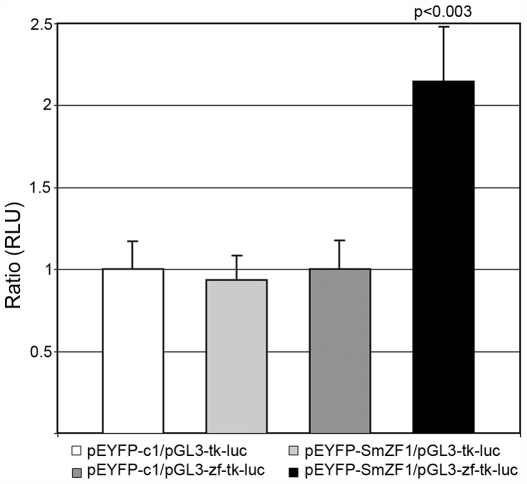
SmZF1 is able to increase luciferase gene expression in COS-7 cells. The plasmids of interest plus the TK-Renilla reporter plasmid were transfected into COS-7 cells using the LipofectamineTM 2000 Transfection Reagent. Each transfection was performed in triplicate, and transfection efficiency was normalized to TK-Renilla luciferase reporter plasmid. Luciferase activity was assayed in cell lysates using a TD20/20 luminometer (Promega) and is indicated as Relative Light Units (RLU). Statistical analysis of the data was carried out with Minitab Version 1.4 using Student's t test, and p values<0.05 were considered as significant. Data is representative of three independent experiments.

Although new vectors which will allow transfection of schistosome cells are under development [40]–[42], it is still not possible to continuously cultivate schistosome cells lineages *in vitro*. Accordingly, some authors describe the use of mammalian cells to study aspects of *S. mansoni* gene regulation processes, such as testing transcription factor activities or mapping promoter regions of genes [28],[43],[44]. Thus, a luciferase system assay in COS-7 mammalian cells expressing YFP-SmZF1 fusion protein was used here to test SmZF1ability to regulate gene transcription.COS-7 cells co-transfected with the expression vector pEYFP-SmZF1 and the construction pGL3-zf-tk-luc, which contains four repetitions of the SmZF1 D1-3DNA binding site and a thymidine kinase minimal promoter upstream of the luciferase coding gene, were able to increase gene transcription by 2-fold (p≤0.003) when compared to negative controls, using the Student's t test (Figure 4). These results suggested that SmZF1 positively affects the transcriptional activity of the minimal thymidine kinase promoter in COS-7 cells.

## Discussion

Schistosomiais is one of 13 neglected tropical diseases that together affect 1 billion people worldwide. The disease is considered the second most socioeconomically devastating parasitic disease, the first being malaria [45]. According to Chirac and Torreele, in the past 30 years the number of drugs which target these neglected diseases is about 1% of all the new chemical entities commercialized by the pharmaceutical industry [46].

*S. mansoni* presents a variety of interesting biological regulatory processes, such as transcriptional control, which can be used to allow its adaptation to the diverse biotic and abiotic environments [8]. Description of genes expressed in a stage- or sex-specific manner may help to elucidate the events used by the parasite to deal with these potentially adverse conditions. In turn, this information may also help to develop suitable vaccines and chemotherapeutic drugs against this organism [7]. As stated in the recent and high quality review on schistosome genomics by Han and colleagues [47], some potential drug targets should include proteins involved in DNA replication, transcription and repair systems. This suggestion is also corroborated by a chemogenomics screening approach described as part of the up-to-date *S. mansoni* genomic analysis, in which the authors used a strategy to find significant matches between parasite proteins and proteins known to be targets for drugs in humans and human pathogens. That study revealed 26 putative *S. mansoni* protein targets and their potential drugs. Of these 26 targets, three proteins are involved in DNA metabolism and two others are involved in chromatin modification (histone deacetylase 1 and 3) [5]. These two examples emphasize the importance of nuclear proteins as potential drug targets.

According to the authors of the *S. mansoni* transcriptome project [48], 2.4% of the categorized ESTs (Expressed Sequence Tags) under the Molecular Function in Gene Ontology (GO) encode transcriptional regulators. A search for conserved domains using the Pfam database in a subset of those transcripts showed that 5% of them consist of zinc fingers of the C_2_H_2_ group [48]. Moreover, most of the 15 Pfam domains found were from proteins involved in either intercellular communication or transcriptional regulation. These findings reinforce the importance of this class of regulatory proteins for *S. mansoni* biology. In addition, using the SAGE approach, Ojopi and colleagues found that 9.7% of the most abundant genes (genes containing more than 500 tags) from *S. mansoni* adult worms comprise those from the nucleic acid binding GO functional category [49].

The present study defines the SmZF1 protein as a *S. mansoni* transcription factor. SmZF1 is a C_2_H_2_ zinc finger protein able to specifically bind to RNA and DNA, but with higher affinity for DNA molecules. Its transcript was identified in the cercaria, egg, schistosomulum and adult worm stages, suggesting its importance as a regulatory protein [35],[36].

To define SmZF1 activity as a transcription factor, we first verified its subcellular localization, since this class of proteins is preferentially located or able to go to the cell nucleus, this import being a central step to regulate gene transcription [50],[51]. *In silico* analyses of the SmZF1 amino acid sequence did not predict any classical potential nuclear localization signal (NLS), but did reveal positively charged amino acids within the zinc finger motifs [35]. It has been demonstrated that zinc finger motifs are sufficient and sometimes essential for nuclear localization of ZF proteins, even without any canonical NLS detected in their amino acid sequences [51],[52]. Moreover, it is well known that small proteins (<40 Kda), like SmZF1, are sometimes able to passively diffuse into the nucleus [50].

Immunohistochemical analysis of the diverse parasite developmental stages demonstrated that SmZF1 was indeed localized in the nucleus of *S. mansoni* cercariae, schistosomula and adult male worms. This confirms previous results obtained by SmZF1 cDNA amplification [35] and reinforces our hypothesis that the protein is a transcription factor. An unexpected result was the lack of detection of SmZF1 protein in adult female worms when assayed by this technique. This differs from available transcriptome data, given the existence of one EST sequence (GenBank accession number BF936884) derived from an adult female worm cDNA library presenting 99% identity with SmZF1. Also, studies using oligonucleotide microarrays in which the SmZF1 sequence was spotted on the slide did not reveal this transcript as being differentially expressed between adult male and female worms [16],[20]. Based on these observations, we performed q-PCR experiments to analyze the SmZF1 mRNA expression. We were not able to detect differences in the levels of SmZF1 transcripts between adult male and female worms, indicating that the *SmZF1* gene is being equally transcribed in adult female as it is in adult male worms. The fact that SmZF1 protein was not detected in adult female worms by immunofluorescence experiments suggests that a post-transcriptional mechanism regulates the gene. It is important to note that, apparently, SmZF1 mRNA levels are low in all parasite life cycle stages, as demonstrated by the number of ESTs matching SmZF1 cDNA present at dbEST (Table S1). Since the SmZF1 protein is highly abundant at the various stages, as verified by immunohistochemistry assays (except for the female adult worm), it can be hypothesized that the protein has a long half life and that the few existing mRNAs may possess a high translational rate. However, this picture might be different for female adult worms, in which the transcript could be less translated or translated in a non-efficient way. As a second hypothesis, the protein in females may present a higher turnover. Future experiments need to be done in order to clarify these points. In a recent study concerning *S. japonicum*, Liu and colleagues analyzed data obtained using either transcriptome or proteome approaches and found several genes with no direct correlation in their expression when comparing these two techniques [53]. The authors explained this fact by limitations in sensitivity of the proteomic technologies they employed, but also highlighted that some transcripts may be relatively stable, persisting throughout several stages and being translated in a shorter window. This could contribute to the discrepancy between the proteomic and transcriptomic data [53].

According to Hokke and colleagues, investigating proteins differentially associated with each sex could reveal important clues concerning the formation of sexually mature schistosomes and consequently leading to the description of novel chemotherapeutic targets acting in the maturation process [54]. Recently, different groups have used a myriad of approaches to describe schistosome genes expressed in a gender- or stage-enriched/specific fashion, emphasizing the importance of identification and characterization of proteins that may be controlling the transcription of these genes [8], [16]–[26]. Moreover, the sex-specific presence of a protein potentially capable of regulating the expression of a large number of other genes, as in the case of SmZF1, becomes undoubtedly important in this context. One molecule, SmLIMPETin appears to modulate gene expression in *S. mansoni* [55]. *SmLIMPETin* gene is less expressed in sexually mature adult females when compared to sexually immature adult females and sexually mature and immature adult males [55]. These observations suggest that the sex-specific expression of a transcription factor may be a common feature involved in the maintenance of this parasite life cycle.

The ability of SmZF1 to activate/repress transcription of a luciferase reporter gene in a cellular context was assessed using COS-7 cells. The first step was to confirm the expression, localization and activity of the fusion protein YFP-SmZF1, used for the assay. YFP-SmZF1 was clearly visualized in the COS-7 cells nuclei using fluorescent microscopy; however, the protein was also visualized as fibrous filaments dispersed at the perinuclear region, probably associated with the cells cytoskeleton. Furthermore, Western blot assays showed the preferential nuclear localization for YFP-SmZF1, although it was also detected to a lesser degree in the cytoplasmic extract fraction. One possible explanation for this finding is that the YFP portion of the fusion protein, considering its larger size, is interfering with the efficiency of its transport to the nucleus. Conversely, recombinant SmZF1-myc tag, a smaller protein, is detected exclusively in the nuclear portion of COS-7 cells.

The second step was to verify the protein activity, i.e., if the recombinant protein YFP-SmZF1 was able to bind to its target DNA. EMSA assays were performed using total COS-7 extracts incubated with the putative SmZF1 best binding sequence, D1-3DNA. The experiments showed that cell extracts expressing the YFP-SmZF1 recombinant protein retarded the migration of D1-3DNA in the gel. When anti-GFP or anti-SmZF1 antibodies were added to the extract-DNA samples, a supershift was observed, confirming the binding of YFP-SmZF1 to its target.

The transcriptional activity of SmZF1 was further tested using a luciferase reporter system. The results showed a 2-fold increase on the luciferase gene expression in COS-7 cells co-transfected with pGL3-zf-tk-luc and pEYFP-SmZF1. The small but significant increase in the luciferase gene expression observed might be due to the absence, in COS-7 cells, of additional proteins that are important for the proper arrangement of the transcriptional complex into the promoter region. Supporting this hypothesis, Emami and colleagues found that a species-specific interaction between TFIID and Sp1 was essential for transcriptional activation, thus suggesting a difference in transcriptional machinery between vertebrates and invertebrates [56]. As for SmZF1, if a binding partner was present, the increase in the transcriptional activation would probably be much more substantial. A similar scenario has been reported for the protein SmNR1 from *S. mansoni*. In a recent work, Wu and colleagues demonstrated that the SmNR1 protein alone is able to activate the transcription of a reporter gene in COS-7 cells, but when another protein already known to interact with it (SmRXR1) is present, this activation increases approximately 2-fold [28]. In order to better characterize SmZF1 action as a transcription factor, future experiments designed to detect the protein binding partners will be necessary.

In addition to the DNA/RNA specific binding ability of SmZF1 [36], the evidence of its nuclear localization, as well as its capacity to activate gene transcription, strongly suggest that SmZF1 is a *S. mansoni* transcriptional regulator. Additional experiments aimed at determining SmZF1 biological role are being performed. Recently our group used RNAi to conduct an *in vitro* phenotypic screening of 32 *S. mansoni* genes, including SmZF1, known to be expressed at the sporocyst stage [57]. In this study, miracidia were cultivated *in vitro*, transformed into sporocysts in the presence of specific dsRNAs and observed during 7 days, in order to evaluate phenotypic changes. The treatment of the *S. mansoni* larvae with SmZF1-dsRNA induced a reduction of 30% on the SmZF1 transcript levels, when assayed by q-PCR. This modest reduction on the transcript levels was accompanied by a shortening at the sporocyst length in two out of three independent experiments, when compared to a negative control in which a GFP-dsRNA was used. These results show that, even with a small reduction at the transcript levels the parasite phenotype was altered, demonstrating the importance of the *SmZF1* gene expression for the parasite larval stage. We believe that the significance of these findings can be extended for the other life cycle stages.

## Supporting Information

Figure S1 Anti-SmZF1 polyclonal antibody recognizes the SmZF1 protein. *S. mansoni* fractionated protein extracts, as well as the recombinant MBP-SmZF1 protein previously cleaved from its MBP portion, were submitted to SDS - PAGE 10% and blotted onto a nitrocellulose membrane. The anti-SmZF1 antibody was used to specifically recognize the protein. TE - total extract, NE - nuclear extract, CE - cytoplasmic extract.
(3.71 MB TIF)

Figure S2 The SmZF1 protein could not be detected in female adult worm body sections by immunohistochemistry assays. Sections of *S. mansoni* fixed female adult worms were incubated with a rabbit anti-SmZF1 antibody, and then with a Cy-5 conjugated anti-rabbit IgG in a solution containing Alexa Fluor 488 phalloidin to stain actin microfilaments. Samples were incubated with propidium iodide to visualize cells nuclei. Fluorescent images were obtained using a 63x oil-immersion objective lens and confocal microscopy (Carl Zeiss LSM 510 META). Images were analyzed with the Zeiss LSM Image Browser software and edited with Adobe Photoshop CS. To help distinguish the individual fluorescence signals, the original fluorescence colors were digitally modified. In the figure, propidium iodide fluorescence is shown in blue and phalloidin-Alexa fluor 488 in green.
(0.67 MB TIF)

Table S1 Frequency of ESTs matching SmZF1 cDNA at the diverse *S. mansoni* life cycle stage libraries at UniGene.
(0.04 MB DOC)
