# Supplementary figures and images for: Molecular Characterization of the Schistosoma mansoni Zinc Finger Protein SmZF1 as a Transcription Factor
Source: PLoS Negl Trop Dis. 2009 Nov 10;3(11):e547. doi: 10.1371/journal.pntd.0000547 (PMC2770324; doi:10.1371/journal.pntd.0000547)

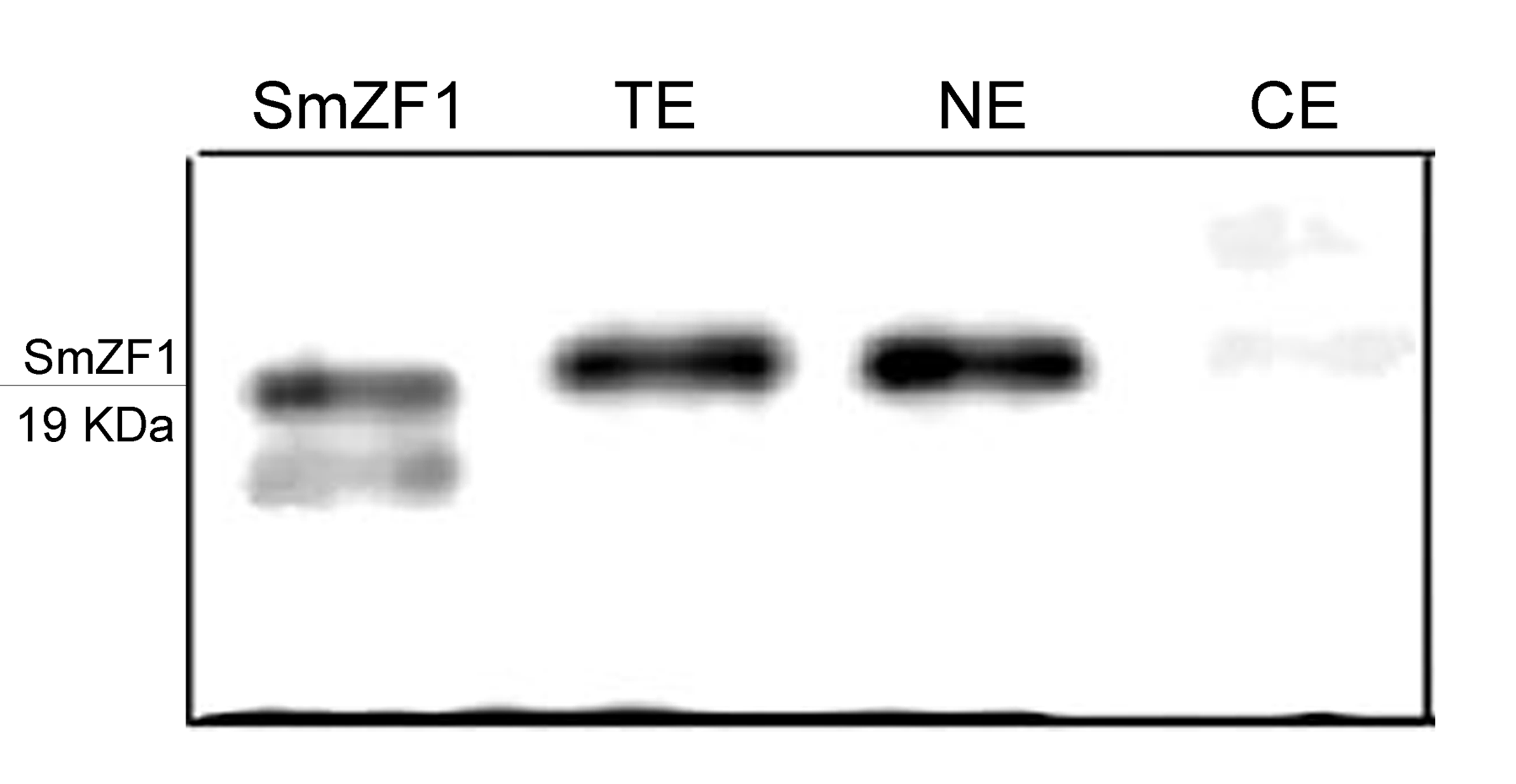

Supplement: Figure S1 — Anti-SmZF1 polyclonal antibody recognizes the SmZF1 protein. S. mansoni fractionated protein extracts, as well as the recombinant MBP-SmZF1 protein previously cleaved from its MBP portion, were submitted to SDS - PAGE 10% and blotted onto a nitrocellulose membrane. The anti-SmZF1 antibody was used to specifically recognize the protein. TE - total extract, NE - nuclear extract, CE - cytoplasmic extract. (3.71 MB TIF) [file pntd.0000547.s001.tif]

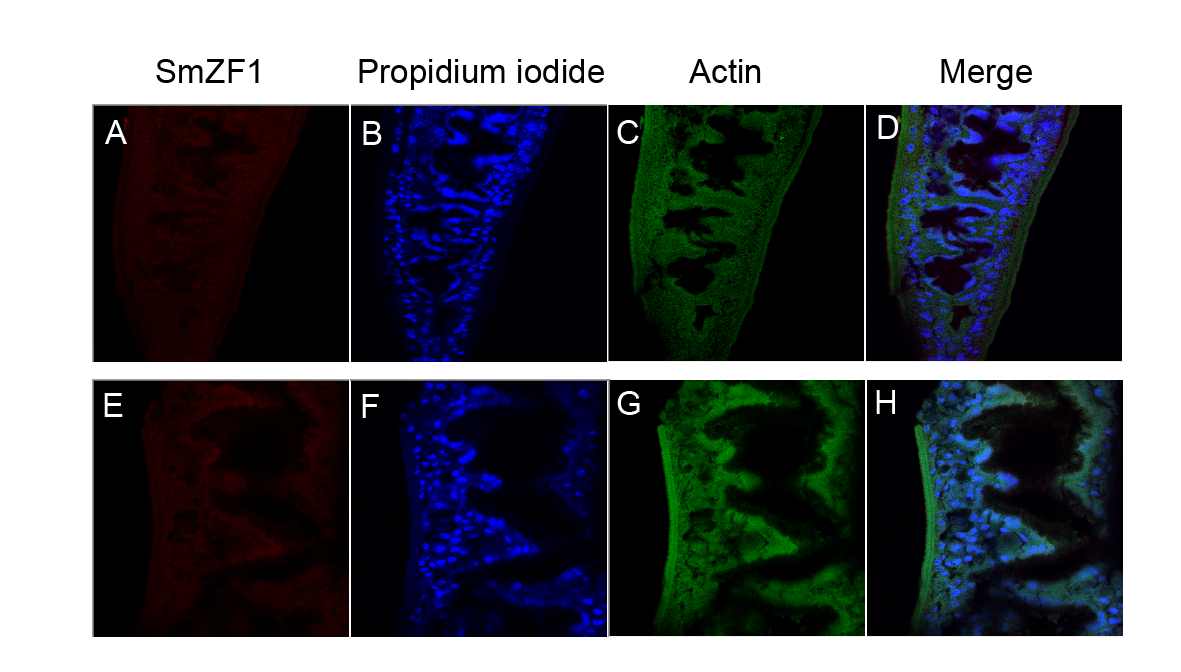

Supplement: Figure S2 — The SmZF1 protein could not be detected in female adult worm body sections by immunohistochemistry assays. Sections of S. mansoni fixed female adult worms were incubated with a rabbit anti-SmZF1 antibody, and then with a Cy-5 conjugated anti-rabbit IgG in a solution containing Alexa Fluor 488 phalloidin to stain actin microfilaments. Samples were incubated with propidium iodide to visualize cells nuclei. Fluorescent images were obtained using a 63x oil-immersion objective lens and confocal microscopy (Carl Zeiss LSM 510 META). Images were analyzed with the Zeiss LSM Image Browser software and edited with Adobe Photoshop CS. To help distinguish the individual fluorescence signals, the original fluorescence colors were digitally modified. In the figure, propidium iodide fluorescence is shown in blue and phalloidin-Alexa fluor 488 in green. (0.67 MB TIF) [file pntd.0000547.s002.tif]
