# Supplementary material for: Molecular Characterization of the Schistosoma mansoni Zinc Finger Protein SmZF1 as a Transcription Factor
Source: PLoS Negl Trop Dis. 2009 Nov 10;3(11):e547. doi: 10.1371/journal.pntd.0000547 (PMC2770324; doi:10.1371/journal.pntd.0000547)
Supplement: Table S1 — Frequency of ESTs matching SmZF1 cDNA at the diverse S. mansoni life cycle stage libraries at UniGene. (0.04 MB DOC) [file pntd.0000547.s003.doc]

**Table S1: Frequency of ESTs matching SmZF1 cDNA at the diverse *S. mansoni* life cycle stages libraries at UniGene**.

| ***S. mansoni* life cycle stage** |  | **Total libraries** | **Total ESTs** | **SmZF1 ESTs** | **SmZF1 frequency** |
| --- | --- | --- | --- | --- | --- |
| **Egg** |  | 98 | 34283 | 3 | 0.0000875 |
| **Miracidium** |  | 104 | 19991 | 2 | 0.0001000 |
| **Sporocyst** |  | 117 | 18028 | 5 | 0.0002773 |
| **Cercaria** |  | 128 | 18561 | 2 | 0.0001078 |
| **Schistosomulum** |  | 145 | 37152 | 7 | 0.0001884 |
| **Adult (total)** |  | 218 | 75810 | 5 | 0.0000660 |
|  | **Female** | 29 | 7851 | 1 | 0.0001274 |
|  | **Male** | 22 | 5259 | 0 | 0 |
| **Unknown** |  | 9 | 2067 | 0 | 0 |
| **Total** |  | 819 | 205892 | 24 | 0.0001166 |
